# Colorectal Cancer in Ukraine: Regional Disparities and National Trends in Incidence, Management, and Mortality

**DOI:** 10.1200/JGO.18.00145

**Published:** 2018-10-24

**Authors:** Nelya Melnitchouk, Galyna Shabat, Pamela Lu, Heather Lyu, Rebecca Scully, Krystle Leung, Molly Jarman, Andrey Lukashenko, Olena O. Kolesnik, Joel Goldberg, Jennifer S. Davids, Ronald Bleday

**Affiliations:** **Nelya Melnitchouk**, **Galyna Shabat**, **Pamela Lu**, **Heather Lyu**, **Rebecca Scully**, **Krystle Leung**, **Joel Goldberg**, and **Ronald Bleday**, Brigham and Women’s Hospital, Harvard Medical School; **Nelya Melnitchouk**, **Rebecca Scully**, and **Molly Jarman**, Center for Surgery and Public Health, Boston; **Jennifer S. Davids**, University of Massachusetts Medical School, Worcester, MA; and **Andrey Lukashenko** and **Olena O. Kolesnik**, National Cancer Institute, National Cancer Registry of Ukraine, Kyiv, Ukraine.

## Abstract

**Purpose:**

The incidence of colorectal cancer (CRC) is increasing worldwide, and the greatest increase is in low- to middle-income countries, such as Ukraine. Better knowledge of epidemiology of CRC in Ukraine is needed to understand how best to decrease the burden of disease.

**Methods:**

The National Cancer Registry of Ukraine (NCRU) was queried for CRC incidence, mortality, stage, and treatment in Ukraine and assessed for regional variation from 1999 to 2015. Joinpoint analysis was used to analyze the trends.

**Results:**

The incidence of colon cancer increased from 10.6 to 13.3 occurrences per 100,000, which provided an average annual percent change (AAPC) of 1.48 (95% CI, 1.3 to 1.7; *P* < .05). The incidence of rectal and anal cancers also increased from 9.9 to 11.5 occurrences per 100,000, which provided an AAPC of 1.0 (95% CI, 0.8 to 1.3; *P* < .05). Mortality remained the same (AAPC, 0.1; 95% CI, −0.3 to 0.2; *P* = .4). The proportion of patients who received cancer-specific treatment increased from 54.6% to 68.5% for colon cancer and from 61% to 74.4% for rectal and anal cancers. Overall, 34.5% of patients with colon cancer and 27.5% of patients with rectal cancer died within a year of diagnosis in 2015. Great regional variations in 1-year mortality and treatment received were identified.

**Conclusion:**

The incidence of CRC in Ukraine is increasing. Despite stable mortality rates, many do not receive cancer-specific treatment, and a large proportion of patients die within a year of diagnosis. These findings illustrate the need to promote establishment of a screening program and to improve access to cancer-specific therapy in Ukraine.

## INTRODUCTION

Colorectal cancer (CRC) is the third most common cancer in the world, and the global burden of disease is expected to increase.^1^ Globally, CRC incidence and mortality rates vary widely and depend on a multitude of factors, such as lifestyle, diet, availability of screening programs, access to care, and gross domestic product per capita.^2^ A few countries, such as the United States, Australia, and Japan, have been able to achieve a decrease in both incidence and mortality.^2^ Some European countries have experienced an increase in incidence but a decrease in mortality, but the majority of the world is facing an increase in incidence and mortality. CRC is treatable if detected at early stages and can be prevented with robust screening programs.^3-5^

Ukraine is a low- to middle-income country with an intermediate incidence of CRC; however, CRC is the third most common cause of malignancy-related death and is an important public health problem. The country is characterized by economic and political instability that contribute to lower life expectancy and health disparities.^6,7^ No screening programs for CRC are in place,^8^ and access to care is limited by financial means.^9-11^ We previously showed that screening for CRC in Ukraine would be cost effective.^12^ However, better knowledge of CRC epidemiology in Ukraine is needed to decrease the burden of disease. The aims of this study are to characterize the epidemiology of CRC in Ukraine from 2009 to 2015; describe the trends in its incidence, mortality, and treatment trends; and examine regional disparities in mortality and treatment received.

## METHODS

### The National Cancer Registry of Ukraine

The National Cancer Registry of Ukraine (NCRU) is a public database that contains detailed population-level data about all of the cancers in Ukraine. The registry encompasses all of Ukraine, includes data about all of the cancers diagnosed in the Ukraine in any given year, and provides 100% coverage of the population as of 2013. The data on Crimea, Donetska, and Luganska Oblast (region) are missing from 2014 to 2015 because of political instability.

The data are rigorously collected on a regional cancer center level. Patients referred to cancer centers are seen within 3 days of referral. The regional cancer center and the hospital or clinic where the patient initially presented are responsible for registering each cancer case. These data from individual hospitals are then reported to regional cancer centers, where the data are then sent to the NCRU. The data about colon cancer are reported separately from the data about rectal cancer, which are pooled with data about anal cancer. This study was approved by Brigham and Women’s Hospital institutional review board.

### Data Collection

The NCRU was queried for CRC incidence, stages at presentation, management, and 1-year and overall mortality rates. The queries covered the 17-year period from 1999 to 2015, and complete data were available from 2000 to 2014. This investigation used the age-standardized rate for world standard population.^13^ The age-standardized values were provided by the NCRU.

### Statistical Analysis

Joinpoint regression analysis was used to analyze the trends in age-standardized incidence, mortality, specialized treatment received, and stage of disease at presentation. Because a significant change in trends was seen, this analysis allowed for identification of the best fit for the joinpoints (or inflection points) using a series of permutation tests with Bonferroni correction. The annual percentage change (APC) and the average annual percentage change (AAPC) were calculated. Joinpoint Trend Analysis software from the Surveillance Research Program of the National Cancer Institute, version 4.5.0.1, was used. In addition, the Pearson correlation coefficient was calculated to assess for correlation between 1-year mortality and treatment received. A *P* value of less than .05 was deemed statistically significant.

## RESULTS

### CRC Incidence and Mortality Trends

**Fig 1:**
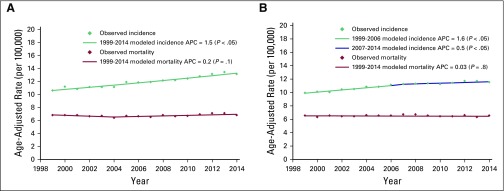
Trends in incidence and mortality of (A) colon cancer and (B) rectal and anal cancers in Ukraine between 1999 and 2015. APC, annual percent change.

During the study period, the incidence of colon cancer increased from 10.6 to 13.3 occurrences per 100,000, which provided an AAPC of 1.48 (95% CI, 1.3 to 1.7; *P* < .05; Fig 1). The incidence of rectal and anal cancers also increased from 9.9 to 11.5 occurrences per 100,000, which provided an AAPC of 1.0 (95% CI, 0.8 to 1.3; *P* < .05). The joinpoint was present in 2007 when the APC changed from 1.64 (95% CI, 1.2 to 2.0; *P* < .05) to 0.43 (95% CI, 0.01 to 0.8; *P* < .05). Colon cancer mortality remained unchanged (AAPC, 0.1; 95% CI, −0.4 to 0.6; *P* = .6; Fig 1). Mortality from rectal and anal cancers also remained the same (AAPC, 0.1; 95% CI, −0.3 to 0.2; *P* = .4; Fig 1).

**Fig 2:**
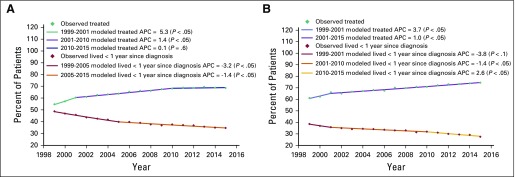
Trends in percentage of patients with (A) colon cancer and (B) rectal and anal cancer in Ukraine who lived less than 1 year from diagnosis and received cancer-specific treatment between 1999 and 2015. APC, annual percent change.

The percentage of patients with colon cancer who died within a year of diagnosis decreased from 48.7% to 34.5% during the study period (AAPC, −2.1; 95% CI, −2.4 to −1.8; *P* < .05). For rectal and anal cancers, the percentage decreased from 38.5% to 27.5% (AAPC, −2.0; 95% CI, −2.5 to −1.4; *P* < .05; Fig 2).

### CRC Treatment Trends

The percentage of patients with colon cancer who received cancer-specific therapy increased from 54.6% to 68.5% during the study period (AAPC, 1.5; 95% CI, 1.2 to 1.7; *P* < .05). For rectal and anal cancers, the percentage increased from 61% to 74.4% (AAPC, 1.3; 95% CI, 1.1 to 1.6; *P* < .05). Of the treated patients, the use of only surgical management decreased from 75.8% to 62.6% for colon cancer (AAPC, −1.2; 95% CI, −1.7 to −1.8; *P* < .05) and from 42.3% to 29.3% for rectal and anal cancers (AAPC, −2.2; 95% CI, −2.8 to −1.6; *P* < .05). The use of combination therapy increased from 19.5% to 29.6% for colon cancer (AAPC, 1.7; 95% CI, 0.7 to 4.1; *P* < .05) and from 33.9% to 43.5% for rectal and anal cancers (AAPC, 1.8; 95% CI, 0.9 to 2.8; *P* < .05).

### Detection at Preventive Visits

The percentage of patients who had colon cancer detected at preventive visits increased from 4.4% to 8.9% (AAPC, 3.6; 95% CI, 1.4 to 5.8; *P* < .05). The percentage who had rectal and anal cancers detected at preventive visits increased from 12.8% to 18% (AAPC, 2.3; 95% CI, 0.5 to 4.0; *P* < .05).

### CRC Stage at Presentation

The percentage of patients with colon cancer who were diagnosed with stages 1 and 2 disease increased from 41% to 50.9% (AAPC, 1.5; 95% CI, 0.7 to 2.2; *P* < .05); with stage 3 disease, increased from 19% to 22.9% (AAPC, 0.7; 95% CI, 0.4 to 1.0); and with stage 4 disease, remained the same at 19% in 1999 and 21.2% in 2015 (AAPC, −0.1; 95% CI, −1.1 to 0.9; *P* = .8). The percentage of patients with colon cancer whose stage was undetermined decreased from 21% to 4.3% (AAPC, −8.7; 95% CI, −8.7 to −12.8; *P* < .05).

The percentage of patients with rectal and anal cancers who were diagnosed with stages 1 and 2 disease remained the same at 56% to 60.7% (AAPC, 0.5; 95% CI, −0.3 to 1.2; *P* = .2); with stage 3 disease, increased from 14% to 18.8% (AAPC, 1.7; 95% CI, 1.0 to 2.5; *P* < .05); and with stage 4 disease, remained the same at 15% to 16.5% (AAPC, 0.3; 95% CI, −0.5 to 1.0; *P* = .5). The percentage of patients with rectal and anal cancers whose stage was undetermined decreased from 15% to 3.6% (AAPC, −4.0; 95% CI, −7.4 to −0.4; *P* < .05).

### Regional Disparity in 1-Year Mortality and Treatment Received in 2013

**Fig 3:**
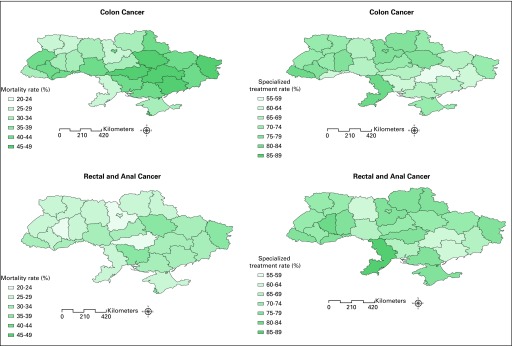
Disparity in mortality and treatment received by region in 2013.

A major difference in 1-year colon cancer mortality exists in different regions in Ukraine; for example, it ranges from 27.7% in Volynska Oblast to 48.1% in Sevastopol. The 1-year rectal and anal cancers mortality ranged from 23.7% in Kirovogradska Oblast to 38% in Sevastopol (Fig 3).

The range of specialized treatment received for colon cancer was also different by region and was wide ranging, from 56.6% in Dnipropetrovska Oblast to 84.6% in Odessa Oblast. The range of specialized treatment received for rectal and anal cancers ranged from 60.8% in Zaporizka Oblast to 85.3% in Odessa Oblast (Fig 3).

**Fig 4:**
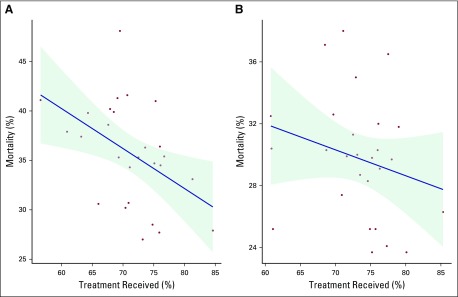
Relationship between 1-year mortality and specialized treatment received for (A) colon cancer and (B) rectal and anal cancer by region in 2013.

There was a moderate linear relationship (*r* = −0.47) between 1-year mortality and specialized treatment received for colon cancer. There was a weak linear relationship for rectal and anal cancers *(r* = −0.24; Fig 4).

## DISCUSSION

In this work, we showed that the incidence of colon, rectal, and anal cancers in Ukraine increased during the study period, whereas mortality remained the same. The percentage of patients who receive cancer-specific treatment is increasing. Although the proportion of patients who die within a year of diagnosis is decreasing (from 48.7% to 34.5% for colon cancer and from 38.5% to 27.5% for rectal and anal cancers), it remains high compared with industrialized nations. Despite the lack of screening, the majority of patients presented with localized, potentially curable disease. We demonstrated that there is a significant disparity in cancer-specific treatment as well as in 1-year mortality between different regions in Ukraine.

Ukraine is lower middle–income country with a post-Semashko health system that has been difficult to reform. Ukraine spends 7.6% of its gross domestic product on health care, and universal free coverage is guaranteed by the Constitution.^14^ In reality, health care is characterized by underfunding, a high proportion of health costs paid out of pocket, and misaligned incentives that have no focus on quality or outcomes.^14^ The major causes of mortality are cardiovascular disease, cancer, and accidents. The burden of noncommunicable diseases is high and continues to increase.^15^ Oncologic care is centralized in regional oncologic centers (ROCs).^16^ Patients with newly diagnosed cancers are referred to ROCs, where all of the oncologic care (including chemotherapy, radiotherapy, and surgery) is provided.

To our knowledge, this is the only study in the literature that describes in detail the epidemiology of CRC in Ukraine. In terms of incidence, this study reports similar results to the data on Belarus and the Russian Federation, countries with similar populations and health system infrastructure, from the GLOBOCAN database. The data quality for mortality was deemed excellent on the basis of the assessment by Mathers et al,^17^ who evaluated data about death registration globally for the WHO. It is encouraging that, despite an increase in incidence, mortality remains the same. This is likely due to an increase in cancer-specific therapy during this timeframe.

The percentage of patients who die within a year of diagnosis remains high despite a significant decrease since 2000. In 2015, 34.5% of patients with colon cancer and 27.5% of patients with rectal cancer died within a year of diagnosis; in contrast, the 5-year mortality in the United States is 35%.^18^ Although the percentage of patients who undergo cancer-specific therapy has increased, a third of patients do not receive any cancer-specific therapy. High 1-year mortality correlates with this finding. There are several potential explanations for this finding. The use of chemotherapy can be costly, or patients might not have access to the care needed to obtain cancer-specific treatment. The quality of cancer-specific therapy might be compromised. A recent survey on the quality of radiotherapy services in post-Soviet countries, including Ukraine, found the need for modernization of equipment and the need for improved staffing, and it noted significant differences in radiotherapy practices compared with the West.^19^ National availability of drugs and out-of-pocket expenditures could be culprits as well.

Interestingly, a large proportion of patients (50.9% with colon cancer, 60.7% with rectal and anal cancers) presented with the disease in localized stages, such as stages 1 and 2. Given that no screening program is in place in Ukraine and that the proportion of patients detected at preventive exams is low, this finding is unexpected. In the United States, only 39% of patients with CRC present with localized disease, and this number is in the setting of a robust screening program.^18^ The NCRU does not describe how CRC is staged in Ukraine, and it is likely that information on staging is not accurate, given that advanced imaging is needed for accurate CRC staging.

We identified significant regional disparity in 1-year mortality and specialized treatment received. There is no literature from Ukraine about regional variation of care to help us understand the reasons for this disparity. Some of the disparity in 1-year mortality for colon cancer can be explained by the number of patients who received specialized treatment, given the moderate linear relationship for colon cancer but not for rectal and anal cancers. The reasons for this disparity are not clear, but it is possible that, because care for rectal and anal cancers is more complicated, more factors are responsible for the disparity.

This study has a number of limitations. The addition of anal cancer, which has different risk factors, biology, and management strategies, might skew the results. However, anal cancer, although its incidence is increasing globally, is still rare: the age-standardized incidence rate is 0.6 per 100,000.^20^ Thus, the impact of anal cancer inclusion should be low. However, the NCRU should separate anal cancer from rectal cancer in the registry, given the increasing incidence of HIV/AIDS, which will likely influence the epidemiology of anal cancer in Ukraine.^21^ Another limitation is that no information included what constitutes cancer-specific therapy in the NCRU. CRC is treated with a multimodality approach, so it would be useful to have more information about the use of different approaches and the percentage of patients who complete the therapy. Stage-specific mortality is not included in the NCRU, which makes it difficult to unravel the exact reasons for the high 1-year mortality.

This is the first study, to our knowledge, to describe the epidemiology of CRC in Ukraine during 17 years and to provide information about regional disparities in management and mortality. This study can be used to guide care for CRC in Ukraine, promote the establishment of a screening program, and support work that improves access to cancer-specific management to decrease regional disparities and improve cancer outcomes. More studies are needed to evaluate the reasons for regional disparities.
